# Expression of Smooth Muscle Calponin in Synovial Sarcoma

**DOI:** 10.1080/13577149977730

**Published:** 1999-09

**Authors:** Hidefumi Ono, Hideki Yoshikawa, Takafumi Ueda, Hisako Yamamura, Ikuo Kudawara, Masayuki Manou, Shingo Ishiguro, Hiroko Funai, Yumiko Koyanagi, Nobuhito Araki, Nobuyuki Hashimoto, Hiroshi Sonobe, Masaharu Tatsuta, Katsuhito Takahashi

**Affiliations:** 1 Department of Orthopaedic Surgery Osaka Medical Center for Cancer and Cardiovascular Diseases 1-3-3, Nakamichi Higashinari-ku Osaka 537-8511 Japan; 2 Department of Medicine Osaka Medical Center for Cancer and Cardiovascular Diseases Osaka Japan; 3 Department of Pathology Osaka Medical Center for Cancer and Cardiovascular Diseases Osaka Japan; 4 Department of Orthopaedic Surgery Osaka University Medical School Suita Japan; 5 Department of Pathology Kochi Medical School Kochi Japan; 6 Department of Gastrointestinal Oncology Osaka Medical Center for Cancer and Cardiovascular Diseases Osaka Japan; 7 Graduate School of Pharmaceutical Science Osaka University Suita Japan; 8 PRESTO Japan Science and Technology Corporation (JST) Japan

## Abstract

*Purpose.* Histogenesis of synovial sarcoma remains controversial and reliable molecular markers for diagnosis are necessary. Expression of basic calponin, a smooth muscle differentiation-specific actin-binding protein, was studied in synovial sarcoma.

*Subjects and Methods.* The basic calponin gene and the gene product were analyzed by reverse transcription PCR analysis (RT-PCR) and immunohistochemistry in 14 synovial sarcomas and a human synovial sarcoma cell line (HS-SY-II).

*Results and Discussion.* Immunoreactivity for basic calponin was detected in the cytoplasm of 6 synovial sarcomas (43% positive). In the basic calponin-positive tumors and the HS-SY-II cells, expression for smooth muscle-specific genes, including basic calponin and SM22α , was detected by RT-PCR, suggesting a lineage relationship between synovial sarcoma cells and smooth muscle-like mesenchymal cells.

*Conclusions.* A subset of synovial sarcomas expressing the basic calponin gene and the gene product were identified. The basic calponin may have potential utility as a novel molecular marker identifying certain synovial sarcomas.


*Sarcoma* (1999) **3**, 107–113

ORIGINAL ARTICLE

### Expression of smooth muscle calponin in synovial sarcoma

HIDEFUMI ONO,[1] HIDEKI YOSHIKAWA,[1] TAKAFUMI UEDA,[1] HISAKO YAMAMURA,[2] IKUO KUDAWARA,[1] MASAYUKI MANOU,[3] SHINGO ISHIGURO,[3] HIROKO FUNAI,[3] YUMIKO KOYANAGI,[3] NOBUHITO ARAKI,[1] NOBUYUKI HASHIMOTO,[4] HIROSHI SONOBE,[5] MASAHARU TATSUTA[6] & KATSUHITO TAKAHASHI[2,7,8]

*Departments of Orthopaedic Surgery[1], Medicine[2], Pathology[3], and Gastrointestinal Oncology[6], Osaka Medical Center for Cancer and Cardiovascular Diseases, Osaka, Japan*
[4]*Department of Orthopaedic Surgery, Osaka University Medical School, Suita, Japan*
[5]*Department of Pathology, Kochi Medical School, Kochi, Japan*
[7]*Graduate School of Pharmaceutical Science, Osaka University, Suita, Japan*
[8]*PRESTO, Japan Science and Technology Corporation (JST), Japan*

**Abstract**
*Purpose.* Histogenesis of synovial sarcoma remains controversial and reliable molecular markers for diagnosis are necessary. Expression of basic calponin, a smooth muscle differentiation-specific actin-binding protein, was studied in synovial sarcoma.
*Subjects and Methods.* The basic calponin gene and the gene product were analyzed by reverse transcription PCR analysis (RT-PCR) and immunohistochemistry in 14 synovial sarcomas and a human synovial sarcoma cell line (HS-SY-II).
*Results and Discussion.* Immunoreactivity for basic calponin was detected in the cytoplasm of 6 synovial sarcomas (43% positive). In the basic calponin-positive tumors and the HS-SY-II cells, expression for smooth muscle-specific genes, including basic calponin and SM22α, was detected by RT-PCR, suggesting a lineage relationship between synovial sarcoma cells and smooth muscle-like mesenchymal cells.
*Conclusions.* A subset of synovial sarcomas expressing the basic calponin gene and the gene product were identified. The basic calponin may have potential utility as a novel molecular marker identifying certain synovial sarcomas.

**Key words**: *calponin, synovial sarcoma, myoepithelium, smooth muscle.*

### Introduction

Synovial sarcomas, which account for approximately 10% of soft tissue tumors, arise most commonly in the para-articular regions in adolescents and young adults.[1] The lineage of differentiation or histogenesis still remains controversial,[2,3] including the synovial,[4] epithelial,[5] neural,[6] and primitive mesenchymal[1] cells in origin. Positive immunostaining for cytokeratin and/or epithelial membrane antigen as well as vimentin has been applied for diagnosis,[1] but histological differential diagnosis may be difficult in the poorly differentiated cases. Therefore, additional genetic or phenotypic markers seem to be necessary to identify synovial sarcoma and predict the biological behaviors of the tumors.

Basic calponin is an actin-, tropomyosin- and calmodulin-binding protein originally isolated from smooth muscle.[7,8] Structural analysis of cDNAs encoding calponin isoforms has revealed the presence of three types of genes with distinct expressional regulation.[7,9,10] Each of the three calponin genes encodes distinct classes of isoforms categorized into basic (p*I* 8 to 10), neutral (p*I* 7 to 8) and acidic (p*I* 5 to 6) calponins on the basis of their isoelectric points. Although the neutral and acidic calponins are expressed in both smooth muscle and non-smooth muscle tissues,[9,11] the basic calponin gene is one of the earliest markers of differentiated smooth muscle cells.[12–15]

Recently, expression of basic calponin has proved not to be restricted solely to smooth muscle cells, and has been detected also in cells that present with certain smooth muscle-like phenotypes, i.e. myofibroblasts and myoepithelial cells expressing a set of other smooth muscle markers.[16,17] With regard to tumors, expression of basic calponin was detected in leiomyomas,[18] leiomyosarcomas,[19,20] and a subset of osteosarcomas.[21]

Correspondence to: Dr Hideki Yoshikawa, Department of Orthopaedic Surgery, Osaka Medical Center for Cancer and Cardiovascular Diseases, 1-3-3, Nakamichi, Higashinari-ku, Osaka 537-8511, Japan. Tel: +81-6-6972-1181; Fax: +81-6-6973-0216; E-mail: yhideki@ort.med.osaka-u.ac.jp

1357-714X print/1369-1643 online/99/020107-07 © 1999 Taylor & Francis Ltd

In the current study, we extended the analysis of basic calponin expression to synovial sarcoma using the tumor samples and a cultured cell line (HS-SY-II).[22] We identified a subset of synovial sarcomas expressing the basic calponin gene and the gene product.

### Materials and methods

### *Tumor tissues*

Tumor samples listed in Table 1 were obtained from primary tumors in 14 patients with synovial sarcoma and in 51 patients with other soft-tissue sarcomas at the Department of Orthopaedic Surgery, Osaka Medical Center for Cancer and Cardiovascular Diseases. The histologic diagnoses and subtypes were established through routine pathologic evaluation according to the published criteria.[1] Diagnosis of synovial sarcoma was confirmed by expression of the SYT-SSX fusion gene resulting from the chromosomal translocation.[23,24] The SYT-SSX fusion gene was detected previously for 8 of 14 synovial sarcomas,[25] and for the remaining 6 synovial sarcomas in this study. Clinical and pathological data of the patients with synovial sarcoma are summarized in Table 2. All synovial sarcomas were considered high grade and deep-seated. Histologic subtypes were as follows; monophasic type: 10 cases, biphasic type: 4 cases. The specimens were fixed in 10% formalin/PBS and embedded in paraffin. Sections of 4 μm thickness were prepared for staining with hematoxylin-eosin, and for immunohistochemical examination.

### *Synovial sarcoma cell line*

A human synovial sarcoma cell line (HS-SY-II),[22] which showed a characteristic chromosomal translocation t(X;18)(p11;q11), was cultured in Dulbecco's modified Eagle's medium (DMEM) (Life Tech., Gaithersburg, USA) supplemented with 10% (v/v) fetal bovine serum (Upstate Biotech., Waltham, USA) and 1% penicillin/streptomycin at 37°C under a humidified atmosphere containing 5% (v/v) $CO_2$. Cells in a logarithmic phase of proliferation were collected and served for detection of mRNAs.

### *Immunohistochemistry*

Monoclonal antibodies against basic calponin (clone hCP) and α-smooth muscle actin (α-SMA)(clone 1A4) were obtained from Sigma Chemicals (St Louis, USA). The specificity of the clone hCP monoclonal antibody to basic calponin isoform was verified as described previously.[11,21] The sections were mounted on poly-L-lysin coated microslides, deparaffinized in xylene, dehydrated through graded alcohol, and immersed in 70% methanol with $H_2O_2$ to block endogenous peroxidase. Antigen retrieval for basic calponin was performed using a 400-W microwave oven (TOSHIBA ERT 330) for 5 min (4 times) in a 10 mM citrate buffer (p*H* 7.0). The sections were incubated with 1% (v/v) goat serum/PBS for 1 h at room temperature, washed in PBS, and incubated with the antibody in 2% (w/v) BSA/PBS overnight at 4°C. They were then washed 5 times with 0.005% (v/v) Tween 20/PBS, followed by incubation with the biotinylated goat anti-mouse IgG (TAGO Immunologicals, Camarillo, USA) in 2% (w/v) BSA/PBS for 1 h at room temperature and avidin–biotin–horseradish peroxidase complex (Vector Laboratories, Burlingame, CA) for 30 min at room temperature. After being washed in 0.005% (v/v) Tween 20/PBS, the final reaction product was visualized with diaminobenzidine (WAKO Chemicals, Osaka, Japan), and the sections were counterstained with hematoxylin. Negative control study with mouse non-immune IgG was included to assess non-specific staining. All assays were scored by three independent observers; staining levels were graded with regard to the number of positive cells in a given tumor sample (++; 50–95% positive cells, +; 10–45% positive cells; –; less than 10% positive cells).

### *Reverse transcription-PCR analysis*

Tumor tissues were frozen immediately after surgical removal and stored at –80°C until extraction of RNA. Total RNA was extracted from tumor tissues using the RNA extraction kit (Nippon Gene, Toyama, Japan). Reverse transcription (RT) of 2 μg of total RNA was carried out using reaction mixture of *Ready-To-Go* You-Prime First-Strand Beads (Pharmacia

**Table 1**. *Immunohistochemical analyses of smooth muscle calponin and α-smooth muscle actin in soft tissue sarcomas*

| | | Calponin positive | | | | α-SMA positive | | | |
|---|---|---|---|---|---|---|---|---|---|
| Diagnosis | No. | 2+ | 1+ | – | rate (%) | 2+ | 1+ | – | rate (%) |
| Synovial sarcoma | 14 | 3 | 3 | 8 | 43 | 0 | 0 | 14 | 0 |
| Malignant fibrous histiocytoma | 11 | 0 | 1 | 10 | 9 | 1 | 4 | 6 | 45 |
| Leiomyosarcoma | 8 | 4 | 2 | 2 | 75 | 5 | 3 | 0 | 100 |
| Liposarcoma | 8 | 0 | 1 | 7 | 13 | 0 | 0 | 8 | 0 |
| Malignant schwannoma | 7 | 0 | 1 | 6 | 14 | 0 | 0 | 7 | 0 |
| Rhabdomyosarcoma | 5 | 0 | 0 | 5 | 0 | 0 | 0 | 5 | 0 |
| Alveolar soft-part sarcoma | 5 | 0 | 0 | 5 | 0 | 0 | 0 | 5 | 0 |
| Dermatofibrosarcoma | 3 | 0 | 0 | 3 | 0 | 0 | 1 | 2 | 33 |
| Angiosarcoma | 2 | 0 | 0 | 2 | 0 | 0 | 0 | 2 | 0 |
| Epithelioid sarcoma | 2 | 0 | 0 | 2 | 0 | 0 | 0 | 2 | 0 |

**Table 2**. *Clinicopathological data of the patients with synovial sarcoma*

| No. | Calponin expression* | Age | Sex | Location | Tumor size** | Histologic subtype |
|---|---|---|---|---|---|---|
| 1 | (++) | 26 | F | leg | 1 | biphasic |
| 2 | (++) | 45 | M | hand | 1 | monophasic |
| 3 | (++) | 45 | M | inguinal | 2 | biphasic |
| 4 | (+) | 20 | F | thigh | 3 | monophasic |
| 5 | (+) | 51 | F | chest wall | 1 | monophasic |
| 6 | (+) | 57 | M | foot | 2 | monophasic |
| 7 | (–) | 61 | M | forearm | 2 | monophasic |
| 8 | (–) | 12 | F | inguinal | 2 | monophasic |
| 9 | (–) | 25 | F | chest wall | 1 | biphasic |
| 10 | (–) | 27 | F | thigh | 1 | monophasic |
| 11 | (–) | 14 | M | popliteal | 2 | monophasic |
| 12 | (–) | 13 | F | buttock | 3 | monophasic |
| 13 | (–) | 37 | F | calf | 1 | biphasic |
| 14 | (–) | 36 | M | buttock | 2 | monophasic |

*(++); 50–95% positive cells, (+); 10–45% positive cells; (–); less than 10% positive cells.
**1; <5 cm, 2; 5–10 cm; 3; >10 cm.

Biotech., Uppsala, Sweden) in the presence of 0.2 μg of the random hexaprimers pd(N)$_6$. After 60 min incubation at 37°C, 0.5 μM of each of the forward and reverse primers, 200 μM of each dNTP mix and 2.5 U of Taq DNA polymerase (Pharmacia Biotech.) were added to 8 μl of the first strand reaction mixture and then, total volume was adjusted to 50 μl with water. The parameters used for the amplification were 30 cycles of denaturation (94°C, 40 s), annealing (60°C, 30 s) and polymerization (72°C, 90 s). Sequences of the selected forward and reverse primers used, and predicted products size were as follows: SYT-SSX, CAACAGCAAGATGCATACCA (forward), CACTTGCTATGCACCTGATG (reverse), 585 bp;[25] basic calponin, GAGTGTGCA-GACGGAACTTCAGCC (forward), GTCTGT-GCCCAACTTGGGGTC (reverse), 671 bp;[21] neutral calponin, CTGCAGAGCGGGGTGGA-CATTGGC, (forward) GCCGGCCTCCTCCT-GGTAGTAAGG (reverse), 519 bp;[21] acidic calponin, GGAAGCGAAGTGCGAGAGACC (forward), CTGTGTGGATCTAATAATCAATGC (reverse), 1061 bp;[21] SM22α, CGCGAAGTGCAGTC-CAAAATCG (forward), GGGCTGGTTCTTCT-TCAATGGGG (reverse), 928 bp;[26] glyceraldehyde 3-phosphate dehydrogenase (GAPDH), CCCAT-CACCATCTTCCAGGA (forward), TTGTCAT-ACCAGGAATGAGC (reverse), 731 bp.[21] The linearity of the PCR products for SYT-SSX, calponins, and SM22α was obtained between 25–30 cycles and for GAPDH between 20–25 cycles. After agarose gel (1%) electrophoresis in the presence of 0.5 mg/ml of ethidium bromide, the PCR products were revealed by UV irradiation, and the image captured, digitized and quantitated by Eagle Eye II Still Video System (Stratagene, La Jolla, USA). Variations in signal intensities between different agarose gels were corrected by using signal intensities of the molecular weight markers in every gel analyzed. Negative results were repeated at least twice.

### Results

### *Immunohistochemical analyses for basic calponin*

Table 1 summarizes the data on immunohistochemistry of basic calponin and α-SMA in soft tissue sarcomas. Among 65 malignant tumors, 6 of 14 (43%) synovial sarcomas showed cytoplasmic immunoreactivity of basic calponin. The basic calponin expression was also detected in 6 of 8 (75%) leiomyosarcomas, 1 of 11 (9%) malignant fibrous histiocytomas (MFHs), 1 of 8 (13%) liposarcomas, and 1 of 7 (14%) malignant schwannomas. The fractions of calponin-positive tumor cells were ranged from more than 90% to as low as 10–20% in synovial sarcoma and leiomyosarcoma samples. Calponin-positive tumor cells in synovial sarcomas showed a spindle cell feature (Fig. 1(a,b)), with no immunoreactivity to epithelial markers such as cytokeratin and epithelial membrane antigen (data not shown). The remaining eight synovial sarcomas lacked immunoreactivity for calponin (Fig. 1(c)). There were no apparent differences in tumor size or histologic subtype between calponin-positive and calponin-negative synovial sarcomas (Table 2).

Calponin-positive leiomyosarcomas tended to show an orderly fascicular pattern and little pleomorphism of tumor cells (Fig. 1(d)). The histology of two calponin-negative leiomyosarcomas was an anaplastic type with less fascicular pattern than the calponin-positive tumors, but they showed immunoreactivity for α-SMA (10–45% positive cells). One MFH, one liposarcoma, and one malignant schwannoma, showed heterogeneous staining for basic calponin (10–20% positive cells). Rhabdomyosarcoma (Fig. 1(e)), alveolar soft-part sarcoma, dermatofibrosarcomas protuberans (Fig. 1(f)), angiosarcoma, epithelioid sarcoma, and the majority of MFHs, liposarcomas, and malignant schwannomas lacked basic calponin immunoreactivity. Notably, in contrast to MFH and

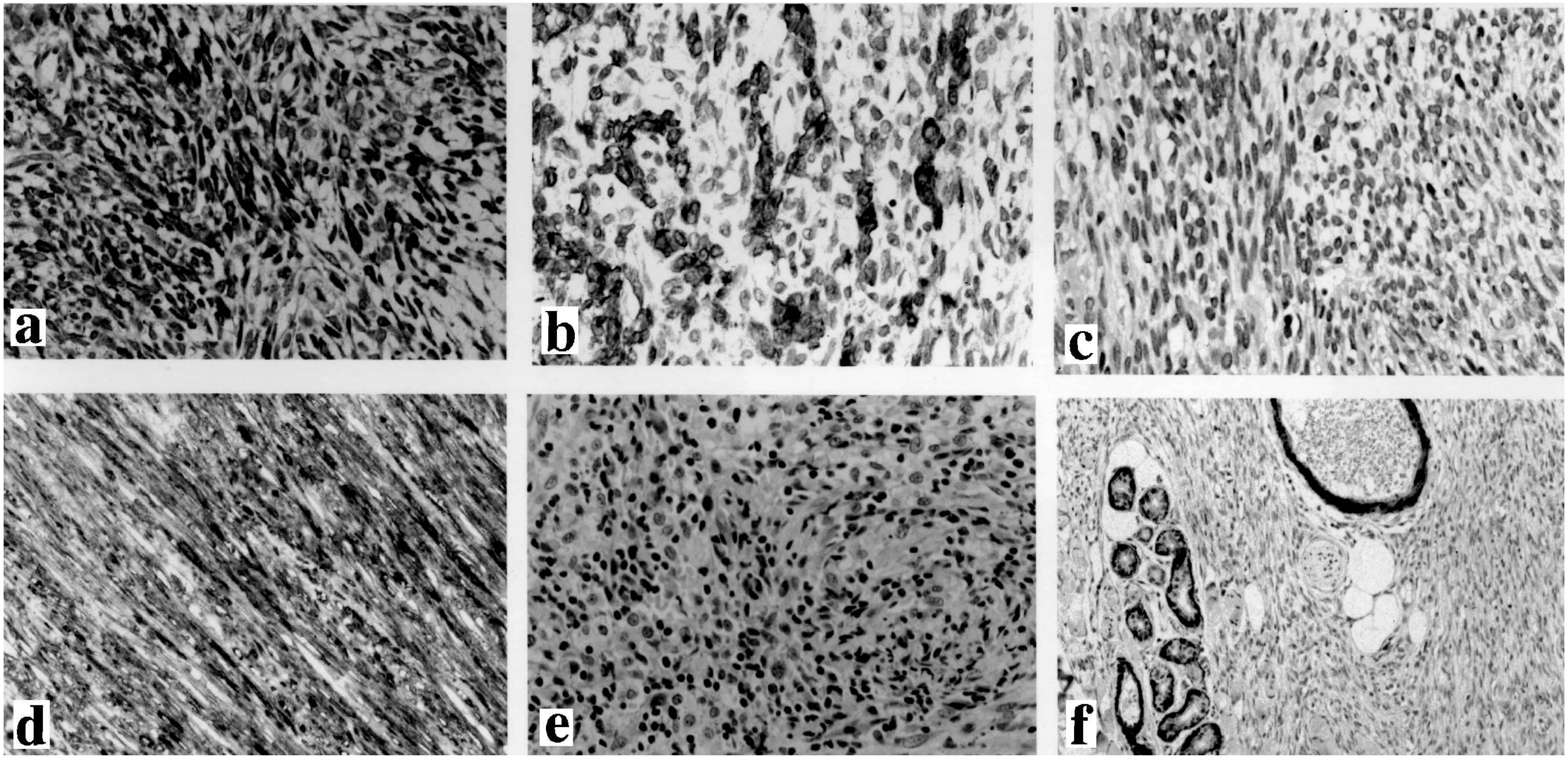

**Fig. 1** *Immunohistochemical detection of basic calponin expression in soft tissue tumors. Immunostaining was localized in the cytoplasm of spindle tumor cells. The sections were counterstained with hematoxylin. (a) Calponin positive synovial sarcoma (diffuse expression pattern), (b) calponin positive synovial sarcoma (focal expression pattern), (c) calponin negative synovial sarcoma, (d) calponin positive leiomyosarcoma, (e) calponin negative rhabdomyosarcoma, (f) calponin negative dermatofibrosarcoma protuberans (tumor cells: negative; smooth muscle cells of a mature vessel and myoepithelial cells of the glands: positive). Original magnification: (a–e); ×50, (f); ×25*

leiomyosarcoma, immunoreactivity for α-SMA was not detected in any calponin-positive synovial sarcomas.

*Expression of calponin isoforms and other smooth muscle markers in synovial sarcomas*

Figure 2 shows the results of RT-PCR analyses for the representative synovial sarcomas with negative and positive immunoreactivity for basic calponin, and for a clonal synovial sarcoma cell line (HS-SY-II). The mRNA transcript of the SYT-SSX fusion gene was detected in both of the two synovial sarcoma tissues. The mRNA transcript of the basic calponin gene was expressed in the case with positive immunoreactivity for basic calponin, but reduced in the case with negative immunoreactivity for basic calponin, indicating a correlation between the intensity of immunohistochemical staining of basic calponin and the expression levels of its mRNA transcript. The HS-SY-II cells also expressed the basic calponin mRNA transcript. The mRNAs for neutral calponin and SM22α were uniformly expressed in the two synovial sarcomas, but acidic calponin mRNA transcript was reduced. In HS-SY-II cells, all calponin isoforms and SM22α were expressed.

### Discussion

Expression of smooth muscle calponin in a subset of synovial sarcomas provides the first evidence that this gene can be expressed in soft-tissue tumors that lack the features of smooth-muscle or myofibroblast differentiation. Synovial sarcomas also expressed the mRNA transcript of another smooth muscle-specific gene, SM22α, suggesting a lineage relationship between synovial sarcoma cells and smooth muscle-like mesenchymal cells.

Recently, we have also detected expression of basic calponin and smooth muscle-specific genes in a subset of human osteosarcomas,[21] suggesting a lineage relationship between smooth muscle-like cells and osteoblasts. Calcification, an unlikely event in other soft-tissue sarcomas, is a well-known feature of synovial sarcoma occurring in approximately 30% of the cases.[27] Furthermore, Milchgrub *et al.* found that synovial sarcoma may produce tumorous osteoid and bone formation rather than the metaplastic bone and proposed a close relationship between osteoblasts and synovial sarcoma cells.[28] The fact that smooth muscle specific genes are commonly expressed in osteosarcoma and synovial sarcoma may support their hypothesis.

Another unique feature of synovial sarcoma is expression of epithelial markers.[1] Expression of both epithelial and smooth muscle markers suggests that histogenesis of synovial sarcoma may be related to myoepithelial cells which present both phenotypes.[16,17] Basic calponin expression holds promise as a new diagnostic marker that enables differential diagnosis of soft tissue sarcomas, particularly in distinguishing certain cases of calponin-producing synovial sarcomas from undifferentiated spindle cell sarcomas. In other words, a basic calponin-positive and α-SMA-negative spindle cell tumor in immunohistochemistry may favor a diagnosis of synovial sarcoma.

Basic calponin is not only a smooth muscle specific gene, but also a cell proliferation-related gene involved in the regulation of cell shape via the actin cytoskeleton.[29,30] Of particular interest is the finding that the N-terminal region of basic calponin shares a characteristic domain structure termed 'calponin homology' or CH-domain with the molecules essential for signal transduction of the Ras *superfamily* of

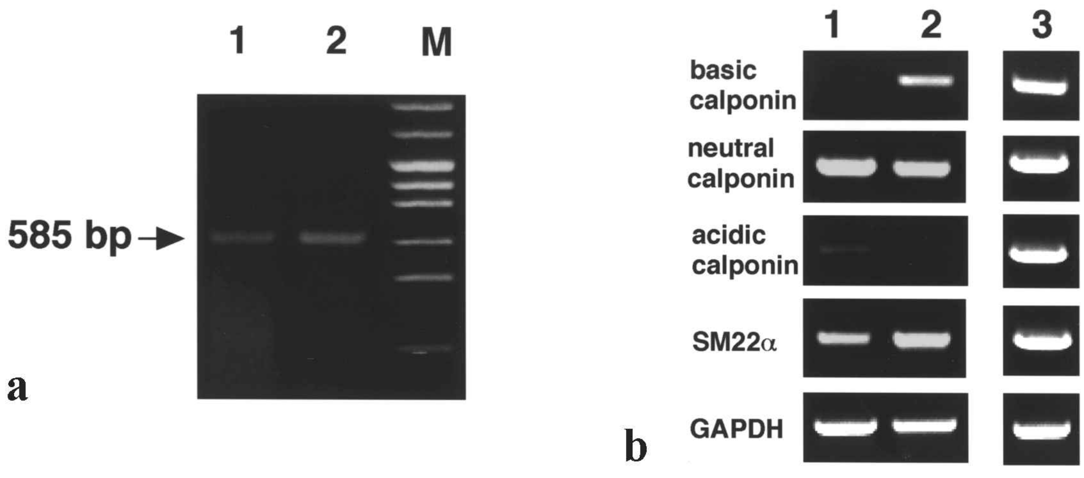

**Fig. 2** *(a) Detection of SYT-SSX fusion gene expression in synovial sarcoma by RT-PCR analysis. Lane 1: synovial sarcoma (Case 8) (basic calponin immunostaining; –. Lane 2: synovial sarcoma (Case 1) (basic calponin immunostaining; ++), PCR product of the fusion gene was 585 bp in size. (b) Detection of basic, neutral and acidic calponins, and SM22α expression in synovial sarcomas by RT-PCR analysis. Lane 1: synovial sarcoma (Case 8) (basic calponin immunostaining; –). Lane 2: synovial sarcoma (Case 1) (basic calponin immunostaining; ++). Lane 3: HS-SY-II cells during proliferation.*

GTPase proteins such as Vav proto-oncogene product and IQGAP1.[31,32] Removal of the N-terminal 67 residues of the Vav protein, which contains a region of the CH-domain, is sufficient to activate the transforming potential of the Vav proto-oncogene product.[33,34] These findings suggest that basic calponin may control actin cytoskeleton, and therefore may exert influence on proliferation, the transformed phenotype and the metastatic potential of the tumor cells. It is also possible that loss of the inhibitory action of calponin on actin–myosin interaction may promote the migration and cytokinesis of the tumor cells, and thus may also influence their proliferation and metastatic potential.

With regard to osteosarcoma patients, a poorer clinical outcome was correlated with loss of basic calponin expression.[21] Therefore, analyses on expression of basic calponin in synovial sarcomas seem to be important for understanding the biological behaviors.

In conclusion, basic calponin expression in a subset of synovial sarcomas may provide us with some leads to solve the histogenesis of these tumors. Further research will examine whether the status of basic calponin expression in synovial sarcomas is a new and independent prognostic factor that may help in the subclassification of these tumor types.

### Acknowledgements

The authors thank Dr David C. Morris for critical reading of the manuscript. This work was supported in part by Grant-in-Aid for Scientific Research from the Ministry of Health and Welfare of Japan, and the Ministry of Education, Science and Culture of Japan.

### References

1 Enzinger FM, Weiss SW. *Soft Tissue Tumors*, 3rd edn. St. Louis: Mosby, 1995, pp. 757–86.
2 Leader M, Patel J, Collins M, Kristin H. Synovial sarcoma: true carcinosarcoma? *Cancer* 1987; 59:2096–8.
3 Miettinen M, Virtanen I. Synovial sarcoma: a misnomer. *Am J Pathol* 1984; 117:18–25.
4 Krall RA, Kostianovsky M, Pathefsky AS. Synovial sarcoma: a clinical, pathological and ultrastructural study of 26 cases supporting the recognition of a monophasic variant. *Am J Surg Pathol* 1981; 5:137–51.
5 Alvares-Fernandez E, Escalona-Zapata J. Monophasic mesenchymal synovial sarcoma: its identification by tissue culture. *Cancer* 1981; 47:628–35.
6 Ichinose H, Powell L, Hoerner HE, Vincent J, Derbes J, Byers JF. The potential histogenetic relationship to the peripheral nerve to synovioma. *Cancer Res* 1979; 39:4270–4.
7 Strasser P, Gimona M, Moessler H, Herzog M, Small JV. Mammalian calponin. Identification and expression of genetic variants. *FEBS Lett* 1993; 330:13–8.
8 Takahashi K, Hiwada K, Kokubu T. Vascular smooth muscle calponin: A novel troponin T-like protein. *Hypertension* 1988; 11:620–6.
9 Appledate D, Feng W, Green RS, Taubman MB. Cloning and expression of a novel acidic calponin isoform from rat vascular smooth muscle. *J Biol Chem* 1994; 269:10683–90.
10 Takahashi K, Nadal-Ginard B. Molecular cloning and sequence analysis of smooth muscle calponin. *J Biol Chem* 1991; 266:13284–8.
11 Masuda H, Tanaka K, Takagi M, Ohgami K, Sakamaki T, Shibata N, Takahashi K. Molecular cloning and characterization of human non-smooth muscle calponin. *J Biochem* 1996; 120:415–24.
12 Duband JL, Gimona M, Scatena M, Sartore S, Small JV. Calponin and SM22 as differentiation markers of smooth muscle: spatiotemporal distribution during avian embryonic development. *Differentiation* 1993; 55:1–11.
13 Gimona M, Herzog M, Vandekerckhove J, Small JV. Smooth muscle specific expression of calponin. *FEBS Lett* 1990; 274:159–62.
14 Miano JM, Olson E. Expression of the smooth muscle cell calponin gene marks the early cardiac and smooth muscle cell lineages during mouse embryogenesis. *J Biol Chem* 1996; 271:7095–103.
15 Samaha FF, Ip HS, Morrisey EE, Seltzer J, Tang Z, Solway J, Parmacek MS. Developmental pattern of expression and genomic organization of the calponin-h1 gene. A contractile smooth muscle cell marker. *J Biol Chem* 1996; 271:395–403.
16 Lazard D, Sastre X, Frid MG, Glukhova MA, Thiery JP, Koteliansky VE. Expression of smooth muscle-specific proteins in myoepithelium and stromal myofibroblasts of normal and malignant human breast tissue. *Proc Natl Acad Sci USA* 1993; 90:999–1003.
17 Savera AT, Gown AM, Zarbo RJ. Immunolocalization of three novel smooth muscle-specific proteins in salivary gland pleomorphic adenoma: Assessment of the morphogenetic role of myoepithelium. *Mod Pathol* 1997; 10:1093–100.
18 Draeger A, Graf AH, Staudach A, North AJ, Small JV. Smooth muscle differentiation in human myometrium and uterine leimyoma. *Virchows Archiv B Cell Pathol* 1993; 64:21–7.
19 Horiuchi A, Nikaido T, Ito K, Zhai Y, Orii A, Tanigucgi S, Toki T, Fujii S. Reduced expression of calponin h1 in leiomyosarcoma of the uterus. *Lab Invest* 1998; 78:839–46.
20 Meyer T, Brinck U. Expression of myogenic marker proteins in human leiomyosarcoma. *APMIS* 1997; 105:793–800.
21 Yamamura H, Yoshikawa H, Tatsuta M, Akedo H, Takahashi K. Expression of the smooth muscle calponin gene in human osteosarcoma and its possible association with prognosis. *Int J Cancer* 1998; 79:245–50.
22 Sonobe H, Manabe Y, Furihata M, Iwata J, Oka T, Ohtsuki Y, Mizobuchi H, Yamamoto H, Kumano O, Abe S. Establishment and characterization of a new human synovial sarcoma cell line, HS-SY-II. *Lab Invest* 1992; 67:498–505.
23 Clark J, Rocques PJ, Crew AJ, Gill S, Shipley J, Chan AML, Gusterson BA, Cooper CS. Identification of novel genes, SYT and SSX, involved in the t(X;18)(p11.2;q11.2) translocation found in human synovial sarcoma. *Nature Genet* 1994; 7:502–8.
24 Kawai A, Woodruff J, Healey JH, Brennan MF, Antonescu CR, Ladanyi M. SYT-SSX gene fusion as a determinant of morphology and prognosis in synovial sarcoma. *N Engl J Med* 1998; 338:153–60.
25 Hashimoto N, Araki N, Kuratsu S, Kudawara I, Yoshikawa H, Uchida A, Ochi T. Clinical relevance of the SYT-SSX fusion gene in synovial sarcoma. *J Musculoskel Res* 1997; 1:111–9.
26 Yamamura H, Masuda H, Ikeda W, Tokuyama T, Takagi M, Shibata N, Tatsuta M, Takahashi K. Structure and expression of the human SM22α gene, assignment of the gene to chromosome 11, and repression of the promoter activity by cytosine DNA methylation. *J Biochem* 1997; 122:157–67.

27 Varela-Duran J, Enzinger FM. Calcifying synovial sarcoma. *Cancer* 1982; 50:345–52.

28 Milchgrub S, Ghandur-Mnaymneh L, Dorfman HD, Albores-Saavedra J. Synovial sarcoma with extensive osteoid and bone formation. *Am J Surg Pathol* 1993; 17:357–63.

29 Jiang Z, Grange RW, Walsh MP, Kamm KE. Adenovirus-mediated transfer of the smooth muscle cell calponin gene inhibits proliferation of smooth muscle cells and fibroblasts. *FEBS Lett* 1997; 413:441–5.

30 Parker CA, Takahashi K, Tao T, Morgan KG. Agonist-induced redistribution of calponin in contractile vascular smooth muscle cells. *Am J Physiol* 1994; 267:C1262–70.

31 Adams JM, Houston H, Allen J, Lints T, Harvey R. The hematopoietically expressed vav proto-oncogene shares homology with the db1 GDP-GTP exchange factor, the bcr gene and a yeast gene (CDC24) involved in cytoskeletal organization. *Oncogene* 1992; 7:611–8.

32 Carugo KD, Banuelos S, Saraste M. Crystal structure of a calponin homology domain. *Nature Struc Biol* 1997; 4:175–9.

33 Coppola J, Bryant S, Toda T, Conway D, Barbacid M. Mechanism of activation of the vav proto-oncogene. *Cell Growth Differ* 1991; 2:95–105.

34 Katzav S, Cleveland JL, Heslop HE, Pulido D. Loss of the amino-terminal helix-loop-helix domain of the vav proto-oncogene activates its transforming potential. *Mol Cell Biol* 1991; 11:1912–20.
